# Half-Yearly Report *of the* Progress *of* Medicine, *from* July *to* December, 1812

**Published:** 1813-01

**Authors:** 


### Medical and Physical Journal.

1 OF VOL. XXIX.] JANUARY, 1813. [NO. 167.

*For the Medical and Physical Journal.*

### HALF-YEARLY REPORT *of the* PROGRESS *of* MEDICINE, *from* JULY *to* DECEMBER, 1812.

(No. VII.)

"The variable composition of man's body, hath made it an instrument easy to distemper, and, therefore, the poets did well to conjoin music and medicine in APOLLO, because the office of medicine is but to tune this curious harp of man's body, and reduce it to harmony."—BACON.

THAT the benefits arising to society by the art of medicine will ever be commensurate to the intelligence, skill, and honor, with which it is exercised, is a position subjected neither to the cavils of ignorance, nor the objections of learning. That men of liberal education, grounded in the elements of natural science, of candid and benevolent minds, neither sharpened by cunning, nor blunted by imbecility, are those best qualified for this important avocation, is no less self-evident. But how may a race of medical practitioners be secured to society, thus qualified in morals and knowledge, to tune and reduce to harmony this "*curious harp of man's body?*" Important indeed to the philanthropist, the philosopher, and the statesman, is this question. It has often been repeated, that, in every age and in every country, respect and honor has been given to the physician. Why then has the Indian alone been sincere in his homage; while in polished society the sarcasm of wit, and the ridicule of buffoonery, have been levelled at a profession, which, mustering in its ranks the most learned among mankind, has frequently been, as a body, the contempt of learning? Because, even where knowledge and civilization are diffused, forgetting the high and honorable post in which he is placed, the medical practitioner titioner has descended to the juggling of the conjurer, to the mean artifice and cunning of the mechanic; and has admitted into his fraternity the unlettered clown, and the designing charlatan.* Let the medical profession be honest to itself, and honorable in the exercise of its functions; let it require earnestly and unreservedly from the legislature a test that shall prove the qualification, both in knowledge and character, of every individual applying to be admitted into its order; then will it be found that the philosopher and the wit, the sage and the savage, will equally reverence a body of men whose avocation it is to remove or soften the "ills that flesh is heir to."

Among the varying and discordant opinions, and the multiform projects, that have arisen for remodelling or meliorating the condition of the medical faculty in the British empire, it becomes peculiarly our duty to notice, as one of the novelties of the present period, the effort now making by the APOTHECARIES to restore their avocation to its former honors and importance. This effort has led to the preceding remarks; and we feel a conviction that, whenever the whole body of the medical profession honestly, liberally, and earnestly, applies to the legislature, the application will produce a statute law, which will remove most occasions for dispute, and prevent the ridiculous and scarcely-honest contentions that are found among physicians, surgeons, and apothecaries. And this conviction we feel, because we cannot suppose the government so blind to the interests of the people, and so negligent of the desires and claims of an extensive and useful class in society, as to refuse what would then be reasonably required. But, while the profession is

---

* We would prove, if it were not already enough known, that physicians of reputation have given certificates to Jew pedlars, livery servants, fortune-tellers, and other vagabonds, by which such persons have been enabled to procure the degree of DOCTOR OF PHYSIC, from universities where no other qualification is required than a written recommendation. Feeling enough of *l'esprit du corps* to advocate always the honor of the profession, we wish such things had not happened; but, having happened, the same feeling compels us to censure their impropriety.

unwisely unwisely divided against itself, can it be expected that the great council of the nation will listen to its propositions.

Unfortunately, however, there does exist, at present, a permanent source of divided interests. The college of Physicians of London has a Charter, considered to be fully equal to all its own particular and individual purposes: the College of Surgeons is under the same circumstance. Can it then be supposed that these bodies will join in any measure that shall have even a remote tendency to loosen, or to alter in anywise, their present privileges? Every measure that touches, however lightly, on their chartered rights, will be resisted, and effectually resisted. Under these existing circumstances, it remains to the *Apothecaries* to look to their own particular condition. But a difficulty here arises; and it must be removed before this body can go to the legislature with a reasonable prospect of success.

Who are the *Apothecaries* of Great Britain? Are they persons practising pharmacy only; or are they persons who join with the pharmaceutic art the practice of physic, surgery, and obstetrics? Those who practise pharmacy alone are few in number, compared with those who exercise all the branches of the profession. Every city, every town, and almost every village, in England and Wales, presents one or more of these general practitioners: but will *they* be legally designated by the term apothecary? If an act be procured purporting to regulate the charges, protect the rights, and define the privileges, of the apothecary only, will it extend beyond the Apothecaries' Company, and not leave the great body of useful practitioners unprotected in its rights, and uncertain of its privileges? We do not believe that the Committee at the Crown and Anchor, who solicit the attention of the Apothecaries of England and Wales to their "*Report relative to the present state of Pharmacy*," mean this restrictive effect; but it is essential that the bill to be founded on this *Report* should unequivocally remove the difficulties which press upon the actual medical practitioners, and that these practitioners be satisfied that it will so operate.

 This

This circumstance being attended to, unfortunately an opposite difficulty arises. If, by extending its view, this committee comprehends, in its projected reform, the practice of surgery; how will it avoid pinging on the corporate rights of the College of Surgeons, and calling forth the opposition of that body? These are points of great intricacy, but of vital importance, and must be disposed of before effectual relief can be obtained.

"Sed motos præstat componere fluctus."

The term Apothecary is too restricted, no definition will make it comprehend the Surgeon and Accoucheur. The term Surgeon belongs exclusively to the College of Surgeons, and cannot be employed on this occasion. The mixed practitioners, as they have been quaintly though aptly called, who make up nearly the whole of the country medical faculty, and a great majority of those in the town, must be designated, legally, by some new term. They neither range under the banners of *Pharmacopola Verus, the True Surgeon, nor the Physician.* They are a body distinct from either, but combining the energies, and exercising the functions, of the three. The mass of the population is submitted to their care, with few exceptions indeed. This circumstance alone, were it not a fact that they have generally a most extensive view of the whole system of medical practice, will mark them as of great consideration to a wise government. If the health of the community is entrusted to them, how essential does it become that they should be qualified for the important charge?

The first object of an act of the legislature, designed to meliorate the condition of the SURGEON-APOTHECARY, or mixed practitioner, should regard, as the *suprema lex*, the health of the people, by instituting a court of examiners, with powers to admit or reject those applying for licences to practice. This, as going directly to the creation of practitioners qualified for the important functions of the Physician, Surgeon, Apothecary, and Accoucheur, should be the foundation of all subsequent proceedings. To make a law to protect the rights and define the privileges of persons sons incapable of performing the functions of the station into which they have obtruded, is an absurdity. It would do worse than suffering the mischief to remain, it would protect and countenance the malpractice of confident ignorance. From among whom this court of examiners should be selected, and with whom the power should be placed, will be matter of detail. The second object of this act should be to define the rights and privileges of the body of practitioners, ascertained to be qualified, and legally authorised to practise, under the primary principle; and to distinctly detail their claims on the public. Keeping these fundamental principles in view, it will not seem difficult to men of business, thoroughly versed in the modes of town and country practice, to form a digest or code, that will meet every difficulty, and subdue every opposition.

Having provided the means for raising a body of practitioners duly qualified for exercising the important trust reposed in them, defined their rights and privileges, and shewn the manner in which they are to be remunerated, the object appears to be accomplished. Not so, we fear. Before this desirable and long-wished-for consummation shall arrive, the committee of Apothecaries will have much to contend with, many jarring interests to reconcile, jealousies to subdue, fears to quiet, and confidence to gain.

We have before observed, that the College of Physicians of London, and the College of Surgeons, feel every attack on their privileges like a wound. They will resist the efforts of the committee with all the power that influence, opinion, and talent, confers. If these mighty hosts oppose, the little phalanx of Apothecaries must be conquered, unless it be reinforced with the strong arm of the country practitioners. When the legions marched to Rome, they gave away the empire.

It has been a painful reflection, that the candid and modest proposal, made by a gentleman at the meeting on the 6th of November, to admit "as a favor, a compliment," some country practitioners into the committee, was rejected. Rejected not, indeed, with the gracefulness of conciliation, but with the cold and mistaken assertion that they would be be useless, because they would not attend ; or with the contradictory opinion, that the larger the committee the less would it accomplish. That country practitioners, when properly incited, will not attend the committee, is not quite so certain, as that it may be too unwieldy for business. Already it is so, with the misfortune of having all its ponderosity on one side. Why should not some of the *household troops* of the present committee, who know not or feel not the auxiliary value and the interests of the country practitioners, retire to make room for those who will support the great cause in the distant provinces ; who appreciate the expectations, the weight, and the resources, of their country brethren ; and who will have the confidence of that important body, the aggregate of which they assist in making.

There is one point above all, which the committee should avoid as the rock that will wreck all its hopes—suspicion of its integrity. Already it has gone abroad that trade rather than science is the object of the committee. If its meetings and its views have yet reached the remote provinces, it may be assured that simultaneous with that diffusion, has been spread the leven of apprehension. The demon of Discord

(——— " malum quo non aliud velocius ullum,
Mobilitate viget, viresque acquirit eundo ;
Parva metu primò, mox sese attollit in auras,
Ingrediturque solo, et caput inter nubila condit ;")

will extend her malignant influence to the remotest nooks of the island : if her horn sounds on the Welsh mountain, its blast will reverberate among the northern lakes.

If we could believe that the committee had a view to the benefit of the Apothecaries' Company only, or principally, and designed to avail itself of the weight of the mixed practitioner to carry this project, we should most heartily despise it, and would endeavor, by every means given to us, to defeat its design. We believe otherwise. We think it honestly laboring for the melioration of the condition of the Surgeon-Apothecary, for the benefit of science, and for the good of society. It is not enough, however, that it be virtuous, it must not be suspected.

And

And here, having an interest in the cause, we may be permitted to suggest the outline of a plan, which will prevent suspicion falling on the committee; a suspicion that would be fatal. COMBINE WITH THE LONDON COMMITTEE A DUE PROPORTION OF COUNTRY PRACTITIONERS,* not taken at random,

* This was written immediately on the meeting of the 6th of November, and resulted from impressions made by the proceedings at that meeting. The meeting on the 20th of the same month shews that the committee itself has become sensible, in a degree, of the untoward sentiments which the proceedings of the 6th were likely to excite; and has anticipated, in part, the observations in this Report. In the first place it has combined with itself nine country practitioners, under the equivocal title of "*honorary members;*" and it has passed a resolution, requesting district meetings of the faculty, and soliciting communications and information from those meetings. These, however, may be considered as but half-measures, calculated rather to excite distrust than to raise confidence. How many will admit that the mixed practitioners of England and Wales are properly represented, or their interests secured, by the "*honorary members*" from Colchester, Hampton, Putney, Hampstead, Plaistow, Richmond, Clapham, and Tunbridge-Wells? Does the committee expect that Yorkshire, with its cities and its populous towns, vying with the metropolis in talent, opulence, and resources, will believe itself to be efficiently represented by the "*honorary member*" of the village of Putney? The proverbial sagacity of our northern brethren will have a keener edge set on it, by this trifling; and the committee will find the guineas of Yorkshire take their road to London with tardy reluctance, until the faculty of that county are fully satisfied that their interests are secured by the proceedings in the metropolis. Without condescending to flatter the London committee for its wisdom and honorable intents, (on this occasion the polished verbiage, and bowing civility of the times, are but poor substitutes for truth and reason,) we must say that, unless it can fairly and fully interest and bring into action the great body of practitioners, designated by the term *Surgeon-Apothecary,* spread over England and Wales, it will not effect its object. And the important weight of this body can only be brought to bear efficiently on the question, by giving it the means of co-operating with the London committee; not merely to have the permission to send observations, suggestions, and money, to it, but TO SEE, TO KNOW, AND TO ACT, IN ITS PROCEEDINGS. *Good God*—(to use the emphatic phrase of one of the most enlightened and efficient members of the London committee)—can it be admitted that Liverpool, Birmingham, Manchester, Exeter, Gloucester, Newcastle, Bristol, Bath, the universities, will confide their interests to four or five villages in Middlesex and Essex; that Tunbridge-Wells shall represent random, however, and by accident. In effecting this, the committee may be on the edge of a dilemma; but the difficulty is not insuperable. Let it propose, and induce by every possible means, county or district meetings of the provincial practitioners throughout England and Wales. Lay the projected plan, both in principle and in detail, as far as it has gone, fully and fairly before these county or district meetings; request these meetings to deliberate coolly and seriously upon it; and solicit from each of them a DELEGATE, the organ of the county or district from whence he comes, and who should, by virtue of that authority, have a seat in the LONDON COMMITTEE.

On this principle, improved and matured by men of competent minds, such an efficient power may be produced as will be irresistible. Founded, primarily, on probity and

---

sent the numerous, powerful, and never-submitting, men of Kent? Common-sense, it is not to be doubted, will shew to the London committee how unequal a project, thus frittered into imbecility, will be to effect the great purpose of its destination.

With every disposition to believe that the London committee means well; with all the amenity towards the honorable men who compose it, that the utmost plenitude of modern refinement can demand, the duty we owe to the faculty at large will not permit us to pass silently over its proceedings. Additional reflection on these proceedings of the London committee has but more firmly fixed our opinion, that DELEGATES from district or county meetings, combining with, and having a right to sit in, that committee, will be the only efficient means of accomplishing the *great and good purpose* to which the body called the Association of Apothecaries of England and Wales, have solicited public attention. Content with having suggested what we believe to be an essentially vital principle to this great object; a principle, which, by producing confidence and unity, will give nerve and energy to all its measures, we shall not enter on the minutia of detail. At the meetings of the Association of Apothecaries we have witnessed the waste of eloquence and of time; and how much elaborate descriptions retard business, and darken the subject they design to illumine: we shall here, therefore, rest our remarks with the assurance made to our country brethren, that the proceedings in London, as far as they come to our knowledge, will be faithfully laid before them, and that every means which the accident of station has given to us, will be employed to further, on sound principles, this great effort to meliorate the condition of the SURGEON-APOTHECARY, AND THE CONSEQUENT GOOD OF SOCIETY.

fairness, fairness; directed in detail, secondarily, by the liberality and honor that ever accompanies true science, and rejecting the tricks, cunning, and littleness, of party, a reasonable hope will arise of the accomplishment, at full, of the expectations of that branch of the medical faculty to which the plan applies. If carried into effect, two points of importance will be determined: 1. The melioration of the *Surgeon-Apothecary* in science and medical skill, for the PUBLIC GOOD. 2. A definition of his rights, and an ascertainment of his claims, proportioned to the present state of society, and providing against the fluctuations that will arise, FOR HIS FAIR REMUNERATION.

For dwelling thus long on this subject, some apology may be required. The sense we have of its importance, and of the good that may result to society from its rational prosecution, must afford that apology. We would induce the Company of Apothecaries to understand and feel the value of the auxiliary aid of the country practitioners; and we would persuade that class of practitioners to see the force that must result from a junction of town and country interests. Above all, we are solicitous to prove that a liberal and honorable conduct in these parties, each to the other, will give a weight and importance to their proceedings, an efficacy to their endeavors, which only can arise from perfect confidence and union.

Since the publication of our former Report, Chemistry has been enriched by Sir Humphrey Davy's first volume of Elements, a new edition of Murray's Chemistry, and by many experiments analytical and synthetical, by the French chemists. Among the former we observe, an examination of a fossil vegetable substance analogous to amber, by M. Destouches—an analysis of the urine of the ostrich, by Fourcroy and Vauquelin—of the rose-colored substance deposited from urine in some fevers, by Vauquelin—of the lymph in the ventricles of the brain, by Haldat:—of the latter an attempt to make blood, or a compound fluid resembling the blood of animals. On the subject of animal chemistry, nothing so elaborate, ingenious, and satisfactory, has presented itself on the continent, as the "*Chemical Researches*

*on the Blood, and some other Animal Fluids,*" by Mr. Brande; and which was given at large in our 165th Number.

Among the chemical facts that have come to our knowledge within the last six months, we notice with peculiar approbation the discovery of a test, of extraordinary delicacy, for Arsenic, by Mr. Hume, of Long Acre. So long since as 1809, Mr. Hume had discovered, and published in the Philosophical Magazine, that a combination of silver with arsenic, afforded the means of detecting the minutest quantity of the latter mineral, when in solution. In our Journal for May and October, 1810, Mr. Hume again noticed the efficacy of this test; but it appears not to have been very generally known, for the authors of a paper in the second volume of the Medico-Chirurgical Transactions, published in the beginning of 1812, when relating a case of poisoning by arsenic, almost assume the merit of the discovery, at least of pointing out, with a superior degree of precision, its application, and an explanation of its rationale. Feeling his right to the discovery, as well as to the explanation of its *modus operandi*, thus infringed, Mr. Hume, in two papers in the preceding volume of this Journal, (pp. 109-284,) justifies his claim, in observations on the statement of Drs. Roget and Marcet. As it appears that Mr. Hume published his discovery in 1809, and gave a further explanation of it in 1810, the claim of Dr. Marcet, for giving this test to the public in 1812, must be very small. The public is indebted, however, to Dr. Marcet, for again bringing a valuable fact before it, and for making it better understood by the consequent controversy.*

In Botany, Materia Medica, and Pharmacy, the present interval may be considered as unusually steril. The first part of a *Flora Virginica*, by Dr. Barton, has appeared in America; a work of great promise, upon a subject worthy of the most patient research, and deserving the utmost acuteness of discrimination. In England, a medical botany,

* The process for making Mr. Hume's test, denominated by him Ammoniaco-nitrate of Silver, will be found in vol. xxviii. p. 286, of this Journal.

in in four thick octavo volumes, has been published by Dr. Stokes. It is no more than a dry catalogue, which may be useful, though possessing but little to interest. In going over the division Pharmacy, in the French Journals some novelties are met with, though of but little importance. Among these, a process for obtaining a pure acetic acid, by M. Lartigue, *Pharmacien à Bourdeaux*—the German method of making English peppermint lozenges—an ætherial camphorated water*—the preparation of *Boules de Mars*, by M. Restat—and some stomachic pills *(pilules digestives)*, by M. Bouriat, give an effect that looks somewhat like a catalogue of nostrums.

As the object and the consummation of all the elementary parts of medical science, and of the auxiliary branches of knowledge, is the attainment of a perfect theory and a successful practice, we are particularly solicitous to collect the facts that belong to this part of our Report; and to give such a detail as may enable our readers to judge of the progress of improvement, and to apply particular discoveries or peculiar modes of practice. If we cannot, every half year, present much of interesting novelty, we are able, we think, to mark a distinctly progressive melioration of the practice of medicine and surgery, and to indicate a practical simplicity pointing to the approach toward perfection in the art.

The great and leading feature of this state of gradual improvement, is a general subsidence of the stimulating practice in fever, and a substitution of cooling evacuants, particularly cathartics and blood-letting, with the free application of cool air, and the cold effusion. In the treatment of *Typhus*, this change has been particularly conspicuous. A paper in the 160th Number of our Journal, by Dr. Pigott, clearly elucidates this point. The cases of *Typhus* there

---

* This "*eau etherée camphorée*" promises to be an useful and elegant preparation of camphor. It is limpid as distilled water, with a mixed taste and smell of camphor and æther; and it blends with distilled water and sirops without difficulty. In each ounce is contained about eight grains of camphor, and eighteen or twenty of æther. In another part of our Journal will be found the formula and process for its preparation.

 given, given, shew how far the practice of purging may be carried, and the results.

The evacuating practice, particularly blood-letting, has been employed with success, it is understood, in a disease hitherto considered to be incurable. If we yet doubt if bleeding, carried to any extent, has cured *Rabies Canini*, when hydrophobia has appeared; it must be admitted there is evidence enough in its favor to lead to its full and energetic employment. Dr. Kinglake had but just favored us with some ingenious observations on the affinity between *Gastritis* and *Hydrophobia*, and the probability of success that would attend the full employment of blood-letting, in each of these diseases, when Dr. Tytler, of Calcutta, communicated a case of hydrophobia actually cured by this remedy. Mr. Shoolbred is also reported to have treated successfully another case of hydrophobia in India, by a great abstraction of blood. In a disease in which all remedies have yet failed, every promise, however slender, is seized on with avidity, and we pass rapidly from despair to expectation. We would not repress this buoyancy, but it must be observed that the evidence is not yet enough decisive. The successful case, under the management of Mr. Tymon, of the 22d dragoons, it is to be feared, was not the specific disease. In this, the abstraction of blood was continued until the pulse was scarcely to be felt in either wrist; but opium, mercurial frictions, and blistering, were also employed. This has necessarily left the curative power of blood-letting less distinct; and it may be a question if the cure (admitting the case to be specific hydrophobia) was effected by either of the substances employed, or by bleeding, singly; or by some happy, though accidental, combination of the powers of the whole. Whatever may be the real fact, enough appears on the face of the relation, to make practitioners look with some confidence, and with great solicitude, to this, as yet, *anceps remedium*. It is to be regretted, that, in the case of hydrophobia, so correctly related by Dr. Pinckard, in the 166th Number of this Journal, the bold abstraction of blood had not been early recommended.

We are more positive as to the success that will arise from the the more extensive employment of a medicine, our knowledge of the efficacy of which is still in its infancy. The instances of the curative effects of Arsenic, in severe and obstinate cases of periodical head-ach, are sufficiently numerous to establish its character in that disease. It is not so well known, however, as a remedy for Epilepsy; and we may be doing a public benefit, by recording the fact of two cases of this disease, of long standing, being cured by the *liquor arsenicalis*. These have occurred within the time allotted to this Number of our Report, and are sufficiently marked to give rise to the hope for further success in this unmanageable complaint;* and the fact, naked as it here stands, may excite to a more extensive employment of this valuable remedy.

A medicinal preparation of gold, which has been noticed in some of the preceding parts of our Journal, has been successfully administered to some syphilitics in London. When we formerly hinted at the charlatanic coloring which Dr. Chrestien gave to his new remedy, a sort of *aurum potabile*, our view was to repress empiricism without discouraging experiment. Our transatlantic brethren cherished an opinion similar to ours, on the sanguine expectation and complacency which the Montpellier physician has indulged for his new remedy. A course of experiments has been commenced at New York, and the result, at present, is in favor of Chrestien's statement. Drs. Seaman and Pascalis are engaged in the investigation; but we are concerned to find, as it has been in this country, that the principal object is to ascertain the antisyphilitic properties of this remedy, without regarding its alleged effects on some forms of *carcinoma*, or on the predisposition to that dreadful malady. For syphilis we have a remedy so certain as almost to deserve the character of a specific; but we know of no material that goes beyond a palliative in cancer.

The attention of the profession has been called to the carbonate of iron, as a remedy for cancer, by the publication

* It is not consonant to this part of our work to record these cases in detail; but we hope, at a future period, to lay them before the public.

of of Mr. Carmichael, of Dublin; and it has been resorted to with a confidence in that disease, to which, we fear, no medicine is yet entitled. This confidence has, probably, arisen from calling that Carcinoma, which should, in truth, have had some other denomination. Considerable illustration has been given to this by the labors of Dr. Gamage, of North America; who has shewn the remedial properties of this carbonate in a disease of the uterus, resembling carcinoma, but which he denominates *ulcerated uterus.* In the last number of the New-England Journal of Medicine and Surgery, Dr. Gamage has given four cases of this disease, with remarks, which will afford some explanation of the curative effects of this remedy in diseases called cancer, and of its failure in genuine carcinoma.

Our readers will possibly recollect some statements in this Report (No. VI.) respecting an alarming disease which had appeared at Blackaton, on Dartmoor, destroying, very suddenly, several persons; and then, as suddenly, subsiding. We are induced again to notice this malady, from the affinity which it seems to have with a disease that has frequently appeared in New England of late years; and which has been described by the American physicians as a "*Spotted Fever.*" Some cases of the American disease, in Dr. Strong's Inaugural Dissertation, at Hartford, in 1810, afford striking marks of the coincidence; and the last Numbers of the New-England Medical Journal tend much to establish the affinity. The American disease is described as sometimes occurring by a sudden stroke resembling apoplexy, and occasionally as that complaint in the form of coma, with partial paralysis or hemiplegia. When the disease begins with local pains, though they shift, they confine themselves in various instances to one side. The head is generally the first part struck with pain; and the pain of other parts mostly tends to the head, the head being rarely or ever free throughout from some pain or other affection; and, when the pain has been very violent, this affection is commonly delirium, often phrenzy. Blindness, or some other peculiarity, and especially a dilated or contracted pupil, commonly attends the eyes; but the other senses, feeling excepted, cepted, seldom suffer. The bodily powers seem dissolved. The chills are commonly general, with paleness. The spirits are depressed, as in the deeper nervous affections. The heart seems distressed; and the pulse, after being first slow, soon quickens; but it is always small and feeble, and sometimes highly variable. The stomach is irritable. There are frequent faintings. The breathing is laborious. Death may occur in ten or twelve hours. Recovery from some of the worst cases has, even in a few days, often only left behind a slight debility. These are some of the prominent features of a fever which has afflicted the state of New England: a comparison with other symptoms of the patients at Blackaton, will establish a remarkable affinity.*

Apoplexy has been treated on lately by Dr. Cheyne with great acuteness and discernment, and his inquiries have led him to adopt blood-letting in all cases of this disease. His opinion is supported by Portal. Both have reprobated the use of emetics; and by both is an unloading of the sanguiferous vessels, resorted to as the effectual remedy. Doubts, however, have arisen among discerning practitioners if there be not a form or variety of apoplexy, which depends for its cause on some state of the stomach; and that the cerebral affection is secondary to, or consequent on, derangement in that organ. Some cases have occurred within our own knowledge which seem to justify this opinion, an opinion that is further supported by the practitioners of America, particularly by Drs. Warren and Fisher, who have lately published cases which clearly point to an oppressed state of the stomach, from food, offending in quantity or quality, as the direct exciting cause of the paroxysm; and that, in these instances, when vomiting occurred spontaneously, or was excited by art, the patient recovered from his alarming state. The utility of emetics in such instances, has the sanction of experience. Dr. Fisher relates a marked case

* In the second Number of the New-England Journal of Medicine and Surgery, for April 1812, it is stated, "that the *Spotted Fever* has, in January, February, and March, invaded various towns of New Hampshire, Vermont, and the district of Maine. This is the seventh year of its appearance in the Northern States and Canada."

of of apoplexy, resulting from an oppressed state of the stomach, in which vomiting rescued the patient from an apparently hopeless condition. The case of paralysis, by Mr. Harrold, in our preceding Number, is much in point as to the utility of emetics under some morbid circumstances of the cerebral organ; the effect, probably, of a state of the stomach not yet sufficiently understood.

In the practical department of medicine, the continental journals have been somewhat more prolific than in the auxiliary and elementary parts. A remedy for Dysentery has been spoken of with much confidence, and experience is said to justify that confidence. Hufeland in particular, who has published the recipe in his Journal de Medecine-pratique, confirms its salutary effects. This remedy is nothing more than pills made of the *Hydrargyro corrosivo albo.* Pharm. Seuc. In Sweden it has been employed some time, and certainly, says Hufeland, with great success. Dr. Clarke, in the preceding volume of this Journal, has suggested a remedy, which, *à priori*, promises to be more useful, and certainly less hazardous than the corrosive sublimate. It is an enema of ipecacuanha: this, we are assured, has been completely successful in a great number of both recent and old cases. The use of a mixture of cream and linseed oil for every species of burn, so highly recommended by *M. le Docteur Rieg*, will hardly be considered as deserving much notice here. The use of belladonna in hooping-cough, as recommended by Hufeland, claims more attention; as does the employment of the *Rhododendrum Chrysanthum*, as a remedy for gout. This active medicine requires to be given with great caution, beginning with very small doses, and proceeding, gradatim, to the maximum. The accidents to be guarded against are excessive vomiting and purging. Those gentlemen who have so ardently been seeking for the active material of the now nearly-forgotten Eau Medicinale d'Husson, would do well to carry their investigations into this family of powerful plants. Not, however, that any thing very new is likely to be offered, perhaps, on the *chrysanthum*, as to its real properties; for, after the investigations of Gmelin, Steller, Koelpin, and Home, who found it serviceable in

 rheumatism rheumatism and gout, and a sedative acting as potently on the heart as digitalis does, reducing the pulsations to 38 in a minute, we must not expect that *Dr. Metternich*, or any modern experimenter, will add much to the catalogue of its virtues. In the relation before us Dr. Metternich asserts that it has radically cured some cases of gout. We are more indebted to *M. Schlesinger* for pointing out the almost specific virtues of the extract of *Lactuca virosa* and *L. scariola*, in spasmodic affections of the chest, particularly Angina pectoris. In doses of two grains, taken frequently, it was found to produce the most beneficial effects. On the preparation of a soporific medicine from the common lettuce *(Lactuca sativa)*, we have been much indebted to the judicious industry of Dr. Duncan (vide his paper on the preparation of the *Lactucarium, &c.* Med. and Phys. Jour. xxviii. 130,) for many useful facts. Of the genus *Lactuca* three species are known to possess the soporific property in varying proportions; but whether differing in quality is not yet ascertained. There is a probability, however, from analogy, that the opium from the *L. virosa* may have properties not precisely like that from the *L. sativa;* it therefore becomes an object, in cases of *Angina Pectoris*, to employ the *L. virosa*, in order to give the experiments of M. Schlesinger a fair and candid trial.

*January* 1*st*, 1813.

